# Household sanitation access and risk for non-marital sexual violence among a nationally representative sample of women in India, 2015-16

**DOI:** 10.1016/j.ssmph.2021.100738

**Published:** 2021-01-23

**Authors:** Georgia Lyn Kayser, Praveen Chokhandre, Namratha Rao, Abhishek Singh, Lotus McDougal, Anita Raj

**Affiliations:** a Division of Global Health, Herbert Wertheim School of Public Health and Human Longevity Science, University of California, San Diego (UCSD), La Jolla, CA, USA; b Center on Gender Equity and Health, Department of Medicine, School of Medicine, UCSD, La Jolla, CA, USA; c Department of Public Health and Mortality Studies, International Institute for Population Sciences, Govandi Station Road, Deonar, Mumbai, India; d Department of Education Studies, Division of Social Science, UCSD, La Jolla, CA, USA; e Department of Public Health & Mortality Studies, International Institute for Population Sciences, Govandi Station Road, Deonar, Mumbai, India; f GENDER Project, International Institute for Population Sciences, Govandi Station Road, Deonar, Mumbai, India

**Keywords:** Sexual violence, Gender-based violence, Shared sanitation, Open defecation, India

## Abstract

**Background:**

Lack of household sanitation, specifically toilet facilities, can adversely affect the safety of women and girls by requiring them to leave their households to defecate alone and at night, leaving them more vulnerable to non-marital sexual violence. This study analyzes the association between household sanitation access and past year victimization from non-marital sexual violence (NMSV) in India.

**Methods:**

We analyzed 74,698 women age 15–49 from whom information on NMSV was collected in India's National Family Health Survey 2015–16 (NFHS-4). We used multivariable logistic regression to test the relationship between women's household sanitation access and recent NMSV experience, controlling for socioeconomics (SES;e.g., age, marital status, caste, wealth, employment), for the total sample and stratified by rural/urban, given lower access to sanitation and lower NMSV in rural contexts.

**Results:**

We found that 46.2% of households in our sample lacked their own private sanitation facilities (58.0% rural; 24.5% urban) and were forced to openly defecate (37.3%) or walk to a shared sanitation facility (8.9%), and 0.45% of women report NMSV in the last 12 months (0.33% rural; 0.68% urban). Our multivariable model indicated no significant association between having private household sanitation facilities and NMSV for the total sample, but stratified analyses indicate a significant association for rural but not urban women. In rural India, those who lack private household sanitation, compared to those with a household toilet, have significantly greater odds of NMSV (AOR = 2.45; p < 0.05). These findings persist after accounting for demographics including age and marital status, socio-economic factors related to marginalization (e.g., caste, wealth), women's employment, and the overall climate of the state.

**Conclusion:**

Findings from this study support prior research suggesting that poor access to sanitation is associated with women's risk for NMSV in rural India. This may be via increased exposure, and/or as a marker for greater vulnerability to NMSV beyond what is explained by other SES indicators. Solutions can include increased access to private household sanitation and more targeted NMSV prevention in rural India.

## Highlights

• Lack of sanitation can adversely affect the safety of women and girls.
• In rural India, many women lack household sanitation and openly defecate.
• Open defecation can leave women and girls vulnerable to harassment and violence.
• We explored this relationship in a nationally representative survey in India.
• Lack of sanitation increased the odds of non-marital sexual violence for rural women.

## Introduction

Globally, 2.3 billion people lack access to basic sanitation (i.e., a latrine/toilet) within their household, and 892 million continue to practice open defecation (WHO UNICEF 2017). Lack of sanitation increases fecal contamination and resultant infectious diseases, including diarrheal diseases, as well as stunting and malnutrition among children (Bartram et al., 2005; Clasen et al., 2014; Dwivedi et al., 2018; Gera et al., 2018; Pickering et al., 2015; Prüss-Ustün et al., 2014; Prüss-Ustün et al., 2019; Schmidt, 2015; Sclar et al., 1982; Ziegelbauer et al., 2012). Combined with low availability of potable water and use of handwashing (i.e., concerns related to WASH- water, sanitation and hygiene), it also contributes to 1.6 million deaths annually; half of these deaths are due to diarrheal disease, particularly among children under five, accounting for 5.3% of all under-5 deaths (Prüss-Ustün et al., 2019). While WASH concerns are a problem globally, there is disproportionate burden in India. Twenty percent of all diarrheal deaths to children under five worldwide occur in India (UNICEF, 2018), where 46% of the country lacks a private household sanitation facility (International Institute for Population Sciences IIPS and ICF, 2017), and 56% of rural residents practice open defecation (WHO UNICEF 2017).

India's public health system has been working to tackle WASH issues for decades, but in 2011, the Government of India accelerated these efforts with the Clean India campaign (known as “Nirmal Bharat Abhiyan”; NBA). NBA's goal is to achieve 100% sanitation access in the country by promoting pour flush twin-pit toilets, which are designed to contain wastes in situ until they are safe to handle (Anuradha et al., 2017, pp. 107–110; WHO UNICEF 2017). Subsequently in 2014, India launched the even more ambitious Swachh Bharat Mission (SBM), mobilizing nearly US$25 billion to eliminate open defecation by 2019, has unprecedented political support and has mobilized nearly $25 billion from Government, the private sector and civil society (Anuradha et al., 2017, pp. 107–110). These efforts do seem to have help increased latrine access in the country (Duflo et al., 2015, p. 21521; Garn et al., 2017; International Institute for Population Sciences IIPS and ICF, 2017), with corresponding decreases in open defecation and unsafe stool disposal seen from 2006 to 07 to 2015–16 (WHO UNICEF 2017). Nonetheless, there remain concerns regarding consideration of social issues that affect latrine use, as well as the social benefits from greater access (Dwivedi et al., 2018; International Institute for Population Sciences IIPS and ICF, 2017). Poorer and socially marginalized groups (e.g., religious and caste minorities) with lower access to basic sanitation facilities are at greater risk for infectious diseases arising from this lower access (Teltumbde, 2014; Lamba & Spears, 2013; Gupta et al., 2016, p. 283; O'reilly & Louis, 2014; Mberu et al., 2016). Girls, too, are more socially and biologically vulnerable to social and health effects from lack of facilities, such an infection, inability to attend schools during menstrual cycles, and risk for sexual harassment and violence (Kayser et al., 2019; Pommells et al., 2018; Saleem et al., 2019; Sclar et al., 1982). Hence, socially marginalized girls are most vulnerable to these concerns.

With recognition of the need for gendered considerations in sanitation, a growing body of research has begun to examine how sanitation might improve physical, mental health, and social well-being of women and girls; these data are used to support health education programs and increase demand and use of household latrines, in India and elsewhere (Caruso et al., 2018; Gonsalves et al., 2015; Pommells et al., 2018; Sahoo et al., 1982; Winter et al., 2019). Qualitative evidence from India suggests that access to private household sanitation may reduce vulnerability to psychosocial stress and sexual harassment, and violence, and improve well-being among women and girls across age groups (Kulkarni et al., 2017; Sahoo et al., 1982). Rural women described exposure to men peeping at them, teasing them, and even sexually assaulting women and girls outside the home, when they seek latrine facilities or a safe environment for defecation outside the home (Sahoo et al., 1982). Urban women also described sexual harassment and abuse experiences in the forms of leering, verbal sexual harassment, having their picture taken without their consent, and men flashing them while using sanitation (Kulkarni et al., 2017). Qualitative research with women in sub-Saharan Africa has yielded similar findings (Pommells et al., 2018; Winter et al., 2018).

Quantitative research on this topic is less available; the sole study we could identify was with a nationally representative sample of women in India which found that open defecation was associated with higher odds of non-marital sexual violence (NMSV) (Jadhav et al., 2016). Unfortunately, this study relies on data that is now more than a decade old, from the third round of India's National Family Health Survey (NFHS-3). Building on this prior research, we tested the association between lack of private household sanitation and this sexual violence outcome using NFHS-4 data. Secondarily, we explored whether these associations differ in urban and rural contexts, an analysis that had not been done before, recognizing that access to sanitation is more likely in urban but NMSV is also more likely in urban settings (International Institute for Population Sciences IIPS and ICF, 2017). Furthermore, open defecation is more common in remote rural areas due to poor roads, distance from urban centers, economic and political marginalization of those in extreme poverty, and insufficient subsidies for sanitation for many poor households (O'Reilly et al., 2017; Jain et al., 2020). Simultaneously, rural areas are also home to socially marginalized groups such as scheduled tribes or castes (Mberu et al., 2016), where exposed girls may face greater vulnerability to sexual violence (Patil, 2016). Our research expands on current knowledge of the risk factors for NMSV, which include but are not limited to civil and ethnic conflict, high unemployment, gender inequality norms, employment rates of women and poverty (Amaral et al., 2015; Kayser et al., 2019; Kethineni et al., 2016). Further research is needed to identify risk factors to help prevent and respond to NMSV in India (Ellsberg et al., 2015).

## Materials and methods

### Ethics

Ethical approval for data collection was provided by the International Institute for Population Sciences (IIPS) Institutional Review Board and the ICF International Institutional Review Board. ICF International is the organization that manages the Demographic and Health Survey (DHS) in India. Ethical exemption for analysis of this deidentified, publicly available data was provided by the University of California San Diego Human Research Protections Program Institutional Review Board.

### Data

This study analyzed the fourth and most recently published round of National Family Health Survey (2015–16), a large-scale demographic household survey conducted by the IIPS under the stewardship of the Ministry of Health & Family Welfare (MoHFW), Government of India. The National Family Health Survey (NFHS) uses a two-stage sampling design in both urban and rural areas. NFHS-4 involved interviews with a total of 60,509 households and 699,686 women aged 15–49 years, across the 640 districts of India. The response rate for women was 97%. See the NFHS-4 National report for further details regarding study design, sampling, tools and protocols (International Institute for Population Sciences IIPS and ICF, 2017).

### Sample

The information on NMSV was collected from women in a sub-sample of 15 percent of households. Only one eligible women age 15–49 per household was randomly selected to answer the questions on non-partner sexual violence (n = 79,729). Trained female enumerators conducted the surveys with women in a private setting without any other family members present, in the primary language of the respondent. We limited our analytic sample to women for whom NMSV data were available, and who were permanent household members for one year or more, as the enumerator assessed sanitation in the current household. Hence, excluded from analysis were n = 5 participants who declined response to the non-marital sexual violence questions, n = 3077 women who had resided in the sampled household residence for less than a year, and n = 1605 women who were non-permanent household members. We also excluded from analysis n = 273 women who were unaware of their caste and n = 71 women who did not respond to the item on fetching time for drinking water. Our final analytic sample was n = 74,698 women (n = 52,475 rural women and n = 22,223 urban women).

### Measures

Our primary independent variable of interest was *household sanitation*, which was assessed via the following question: “What kind of toilet facility do members of your household usually use?” Responses that indicated the presence of household “flush toilet” (e.g. flush to piped sewer system, flush to septic tank, flush somewhere else, or flush to don't know where) or any “pit latrine” (e.g. ventilated pit latrine, pit larine with slab, pit latrine without slab, twin pit/composting toilet, dry toilet) that were not shared were coded as “household sanitation.” This definition is consistent with our focus on infrastructure accessibility for women and girls, not the national and international standard for basic or improved household sanitation (WHO UNICEF 2017) as we are interested in NMSV risks for women and girls when they are forced to find a place to defecate alone or walk to a shared sanitation facility. Our definition of “household sanitation” includes any private household sanitation facility that was not shared with another household (International Institute for Population Sciences IIPS and ICF, 2017; WHO UNICEF 2017). Responses indicating “no facility/uses open space or field” or any “shared sanitation with other household(s)” were coded as “no household sanitation” as they are not used solely by the household members and we hypothesized greater risk for NMSV for women and girls.

Our primary outcome variable of interest was NMSV, which was based on a single item asking: “In the last 12 months, has anyone (other than (your/any) husband, if applicable) physically forced you to have sexual intercourse when you did not want to?” Responses were Yes or No.

Covariates included in the models were measured at the individual level (demographics), household level (socio-economic status), community level (rural/urban residence), and employment level (women's economic participation/employment). The individual-level demographics included woman's age, education, and marital status. Regarding marital status, we combined unmarried women with women whose gauna had not been performed. Gauna is a tradition practiced primarily in the northern states of India in which a young bride lives with her parents until a menstruation ceremony is performed, after which time the bride goes to live with her husband. As such, girls for whom gauna has not been performed are not exposed to the physical and social risks and benefits associated with marriage.

Household level characteristics included religion, caste, wealth index, and time required to fetch household drinking water. We calculated the wealth index using a principal components analysis of household assets and characteristics using standard methods for the NFHS (Ellsberg et al., 2015; International Institute for Population Sciences IIPS and ICF, 2017). We conducted a principle component based factor analysis using all standard items excluding sanitation for the country, and then for urban and rural areas. From the newly calculated composite wealth indexes, we constructed a percentile distribution and categorized households into quintiles demarcating poor, middle, and rich. We categorized time required to fetch drinking water as being less than 30 min or (categorized as ‘near household premises’) 30 min or more (categorized as ‘away from household premises’).

Our community-level variable was rural versus urban (International Institute for Population Sciences IIPS and ICF, 2017). At the employment-level, women's economic participation, was based on the question: “Have you done any work in the past 12 months?” If participants replied yes and reported that they were paid either in “cash” or “cash and kind,” they were categorized as employed.

### Data analysis

Prevalence data were calculated for all variables, including prevalence of NMSV in the past 12 months, for the total sample and by the rural and urban subsamples. Subsequently, we conducted a series of logistic regression models to understand the association between household sanitation and NMSV in the past twelve months. Our initial model was a simple regression with no covariates (Model 1). We then conducted multivariable analysis to adjust for covariates at each of our levels of interest: individual (age, education, marital status), household (religion, caste, wealth, and time required to fetch drinking water), community (rural/urban), and employment. Model 2 included individual level characteristics. Model 3 included individual and household level characteristics. Model 4 included individual, household and community level characteristics. Model 5 included individual, household, community and employment level characteristics. We then disaggregated the data by urban and rural and ran simple and multivariable regression analyses = Models 1, 2, 3, and 5- for urban and rural strata, separately. We then conducted a state fixed effects analysis to eliminate the risk of bias due to variables that vary across states. A goodness of fit test, (the likelihood-ratio test) was used to compare the models and to understand if the explanatory variables in each successive model fit significantly better than the previous model.We applied appropriate sampling weights to all analyses, which included the weight for the random selection of one woman per household. The detailed strategy for calculation of weights is given in the NFHS-4 report (International Institute for Population Sciences IIPS and ICF, 2017). All data analyses were conducted using STATA 15.1, and were adjusted for survey design.

## Results

Study findings document that 46.2% of women have no household sanitation facility; more specifically, 8.9% rely on a shared facility and 37.3% have no sanitation facility access at all from their home, as shown in Table 1 [Samples sizes for all variable categories are provided in Supplemental Material 1**]**. A lack of household sanitation is more prevalent for rural households than urban households (58.0% and 24.5%, respectively).

**Table 1:** Sample characteristics for total sample and by rural and urban subsamples, NFHS-4 2015–16 (India n = 74,698, rural n = 52,475); urban n = 22,223.

Background characteristics	Sample size					
	Total		Rural		Urban	
	wt n	i = wt %	wt n	i = wt %	wt n	i = wt %
***Household Sanitation facility***						
*Yes*	39,532	53.79	20,033	42.01	19,500	75.50
*No*	27,386	37.30	24,661	51.70	2,724	10.60
*Shared sanitation*	6,570	8.90	2,987	6.30	3,583	13.90
***Age***						
15-24	24,246	32.99	16,284	34.15	7,962	30.85
25-34	22,324	30.38	14,314	30.02	8,010	31.04
35-49	26,918	36.63	17,083	35.83	9,835	38.11
**Education**						
Up to primary	29,310	39.88	22,578	47.35	6,732	26.09
Secondary	34,703	47.22	21,436	44.96	13,268	51.41
Higher	9,475	12.89	3,667	7.69	5,807	22.50
***Marital Status***						
Never married	17,062	23.22	10,565	22.16	6,497	25.17
*Ever married women*	56,427	76.78	37,115	77.84	19,311	74.83
***Wealth quintile (without sanitation variable)***						
Poor	19,559	26.62	17,738	37.20	1,821	7.06
Middle	24,855	33.82	17,895	37.53	6,961	26.97
Rich	29,074	39.56	12,048	25.27	17,025	65.97
***Caste***						
Scheduled castes	14,375	19.56	10,323	21.65	4,052	15.70
Scheduled tribes	6,739	9.17	5,557	11.65	1,182	4.58
Other backward class	32,459	44.17	21,024	44.09	11,435	44.31
No caste/tribe	19,915	27.10	10,777	22.60	9,138	35.41
***Religion***						
Hindu	58,870	80.11	39,551	82.95	19,319	74.86
Muslim	10,544	14.35	5,641	11.83	4,903	19.00
Others	4,073	5.54	2,488	5.22	1,585	6.14
**Household water facility**						
Within or nearby premises	68,665	93.44	43,648	91.54	25,016	96.94
Away from premises	4,823	6.56	4,032	8.46	791	3.06
***Working status of women***						
*No*	55,165	75.07	35,381	74.20	19,783	76.66
*Yes*	18,323	24.93	12,300	25.80	6,024	23.34

We also found that 0.45 percent of Indian women reported experiencing NMSV in the past 12 months (0.68% in urban areas and 0.33% in rural areas). While this prevalence is low, possibly due to under-reporting, it nonetheless indicates that more than 4 in every 1000 women, and almost 7 in every 1000 urban women, has experienced NMSV in the past 12 months. Further, in urban settings, more than 1 in every 100 young women 15–24 and working women of any age has experienced NMSV in the past year. Additionally, 1 in 40 urban scheduled tribe women and more than 1 in 30 urban women with no caste/tribe have been victims of NMSV in the past 12 months.

In Table 2 we provide the prevalence of NMSV by sanitation facility, as well as selected socioeconomic and demographic characteristics. While there is a significant association between lacking household sanitation and recent victimization from NMSV, this association appears to hold true for rural but not urban women. In rural India, recent NMSV was reported by 0.18% of rural women with a private household sanitation facility but 0.44% of rural women with no household sanitation facility (p < 0.05). In urban areas, we found that NMSV is more common among secondary and higher educated urban women and working women. In rural areas, we found NMSV to be more common among rural women with no education, and working women.

**Table 2:** Prevalence of NPSV for India, rural and urban by socioeconomic and demographic characteristic (2015–16), N = 74,698.

Characteristic	India				Rural			Urban		
	*%*	CI	chi-2		%	CI	chi-2	%	CI	chi-2
***Household sanitation facility***										
*Yes*	0.44	[0.37,0.50]		*chi2* = *8.06; p* = *0.005*	0.18	[0.12,0.23]	*chi2* = *12.33; p* = *0.000*	0.71	[0.58,0.83]	*chi2* = *0.001; p* = *0.971*
No	0.46	[0.39,0.53]			0.44	[0.36,0.51]		0.57	[0.38,0.76]	
***Age***										
15-24	0.56	[0.45,0.66]		*chi2* = *5.29 p* = *0.071*	0.32	[0.22,0.41]	*chi2* = *3.42; p* = *0.181*	1.04	[0.77,1.31]	*chi2* = *3.04 p* = *0.219*
25-34	0.39	[0.31,0.46]			0.32	[0.24,0.40]		0.50	[0.34,0.65]	
35-49	0.41	[0.33,0.48]			0.34	[0.25,0.42]		0.52	[0.37,0.67]	
**Education**										
Up to and including primary	0.33	[0.26,0.39]		chi2 = 1.04; p = 0.594	0.38	[0.28,0.46]	chi2 = 0.706; p = 0.702	0.18	[0.07,0.28]	chi2 = 5.02; p = 0.081
Secondary	0.53	[0.45,0.60]			0.28	[0.21,0.34]		0.93	[0.75,1.10]	
Higher	0.54	[0.38,0.69]			0.33	[0.13,0.51		0.67	[0.43,0.90]	
***Marital status***										
Never Married	0.57	[0.43,0.69]		chi2 = 11.15; p = 0.001	0.37	[0.24,0.50]	chi2 = 10.67; p = 0.001	0.88	[0.60,1.15]	chi2 = 1.31; p = 0.251
*Ever married women*	0.42	[0.36,0.46]			0.32	[0.26,0.36]		0.61	[0.49,0.72]	
***Caste***										
Scheduled castes	0.41	[0.29,0.51]		*chi2* = *5.08; p* = *0.166*	0.46	[0.32,0.59]	*chi2* = *7.27; p* = *0.064*	0.28	[0.09,0.45]	*chi2* = *5.02; p* = *0.170*
Scheduled tribes	1.03	[0.86,1.19]			0.50	[0.37,0.63]		3.52	[2.82,4.22]	
Other backward class	0.27	[0.20,0.33]			0.26	[0.18,0.33]		0.29	[0.17,0.39]	
None of them	0.58	[0.47,0.68]			0.25	[0.1,0.33]		0.97	[0.73,1.19]	
***Religion***										
Hindu	0.47	[0.41,0.52]		*chi2* = *4.23; p* = *0.120*	0.34	[0.28,0.40]	*chi2* = *4.36; p* = *0.113*	0.73	[0.59,0.86]	*chi2* = *1.14; p* = *0.565*
Muslim	0.31	[0.20,0.41]			0.19	[0.08,0.29]		0.45	[0.24,0.65]	
Others	0.52	[0.37,0.66]			0.42	[0.26,0.57]		0.69	[0.37,0.99]	
**Household water facility**										
Within or nearby premises	0.44	[0.39,0.49]		*chi2* = *11.13; p* = *0.001*	0.31	[0.26,0.36]	*chi2* = *5.03; p* = *0.025*	0.68	[0.56,0.78]	*chi2* = *12.57; p* = *0.000*
Away from premises	0.56	[0.36,0.75]			0.53	[0.32,0.72]		0.71	[0.09,1.33]	
***Wealth quintile (without sanitation variable)***										
Poor	0.49	[0.39,0.57]		*chi2* = *7.77; p* = *0.021*	0.50	[0.40,0.59]	*chi2* = *8.95; p* = *0.011*	0.37	[0.09,0.64]	*chi2* = *2.11; p* = *0.348*
Middle	0.42	[0.33,0.49]			0.24	0.17,0.31]		0.87	[0.64,1.09]	
Rich	0.46	[0.37,0.53]			0.21	[0.12,0.28]		0.63	[0.49,0.76]	
***Working status of women***										
*No*	0.33	[0.28,0.37]		*chi2* = *42.51; p* = *0.000*	0.22	[0.17,0.26]	*chi2* = *33.25; p* = *0.000*	0.54	[0.42,0.64]	*chi2* = *9.70; p* = *0.002*
*Yes*	0.81	[0.68,0.94]			0.65	[0.51,0.78]		1.14	[0.84,1.42]	
***Total prevelance rate of NMSV***	0.45	[0.40, 0.49]			0.33	[0.27, 0.37]		0.68	[0.56,0.78]	

In Table 3, we present the logistic regression analyses of the relationship between Indian women's household sanitation facility and NMSV. We found no significant association between women's access to a household sanitation facility and experience of NMSV in either our unadjusted or multivariable models with the all India sample. In Table 3, when controlling for state fixed effects, we also found no significant association. However, in the full model (Model 5), when controlling for individual, household, and community predictors, as well as the climate of the state, employed women, non-Muslim or Hindu women, and women with water that is greater than 30 min roundtrip from the household were significantly more likely to report recent NMSV.

**Table 3:** Results of adjusted logistic regression model showing association between Indian women's household sanitation facility and their experience of NPSV within the last twelve months (2015-16], N=74698.

	Model-1		Model-2		Model-3		Model-4		Model-5	
	OR		AOR		AOR		AOR		AOR	
***Predictor***										
***Household sanitation facility***										
Yes®										
No	1.05	1.414*	1.203	1.391*	1.128	1.316	1.041	1.195	1.147	1.209
	[0.48,2.26]	[1.04,1.90]	[0.55,2.60]	[1.01,1.90]	[0.47,2.66]	[0.95,1.82]	[0.47,2.29]	[0.83,1.71]	[0.54,2.40]	[0.84,1.73]
***Individual level predictors***										
***Age***										
15-24			1.077	1.035	1.1	1.031	1.405	1.225	1.531	1.235
			[0.38,3.05]	[0.65,1.63]	[0.39,3.09]	[0.65,1.63]	[0.53,3.71]	[0.77,1.95]	[0.56,4.16]	[0.77,1.96]
25-34			0.847	1.075	0.853	1.077	0.919	1.127	0.936	1.129
			[0.46,1.55]	[0.76,1.51]	[0.47,1.54]	[0.76,1.52]	[0.52,1.62]	[0.79,1.59]	[0.53,1.64]	[0.79,1.59]
35-49®										
**Education**										
Up to and including primary			0.582	1.362	0.585	1.375	0.583	1.349	0.659	1.378
			[0.16,2.00]	[0.77,2.38]	[0.17,1.94]	[0.77,2.44]	[0.19,1.78]	[0.73,2.46]	[0.21,2.04]	[0.75,2.52]
Secondary			0.937	1.284	0.953	1.319	1.04	1.464	1.142	1.488
			[0.32,2.70]	[0.76,2.15]	[0.33,2.73]	[0.78,2.21]	[0.39,2.74]	[0.86,2.49]	[0.41,3.12]	[0.87,2.53]
Higher®										
***Marital status***										
Never Married*®*										
Ever married women			0.955	0.524**	0.957	0.526**	1.052	0.573*	1.111	0.575*
			[0.48,1.88]	[0.33,0.81]	[0.49,1.85]	[0.34,0.81]	[0.55,1.98]	[0.37,0.88]	[0.57,2.14]	[0.37,0.88]
***Household level predictors***										
***Caste***										

Scheduled castes					0.685	0.916	0.621	0.851	0.629	0.852
					[0.34,1.35]	[0.58,1.42]	[0.31,1.20]	[0.54,1.33]	[0.32,1.22]	[0.54,1.33]
Scheduled tribes					1.805	0.984	1.534	0.857	1.647	0.865
					[0.81,3.98]	[0.59,1.63]	[0.63,3.71]	[0.51,1.43]	[0.65,4.11]	[0.51,1.45]
Other backward class					0.461**	0.685	0.448**	0.684	0.461**	0.686
					[0.25,0.82]	[0.45,1.02]	[0.24,0.80]	[0.45,1.02]	[0.25,0.82]	[0.45,1.03]
Others®										
***Religion***										
Hindu®					0.688	0.758	0.727	0.779	0.651	0.757
Muslim					[0.19,2.42]	[0.44,1.28]	[0.20,2.59]	[0.45,1.32]	[0.17,2.43]	[0.44,1.29]
					0.869	1.839*	0.855	1.813*	0.88	1.809*
Others					[0.43,1.72]	[1.02,3.31]	[0.42,1.70]	[1.01,3.25]	[0.44,1.73]	[1.01,3.24]
										
**Household water facility**										
Within or nearby premises®										
Away from premises					1.145	1.691*	1.095	1.590*	1.185	1.603*
					[0.57,2.27]	[1.10,2.58]	[0.56,2.12]	[1.03,2.43]	[0.63,2.20]	[1.04,2.45]
**Wealth quintile (without sanitation variable]**										
Poor							1.051	1.154	1.614*	1.212
							[0.60,1.83]	[0.72,1.83]	[1.05,2.46]	[0.75,1.95]
Middle							0.912	0.812	1.166	0.836
							[0.49,1.67]	[0.54,1.21]	[0.58,2.33]	[0.55,1.25]
Rich										
***Working status of women***										
No®										
Yes							2.671***	2.213***	2.583***	2.211***
							[1.85,3.84]	[1.64,2.97]	[1.78,3.74]	[1.64,2.97]
**Community level predictor**										
**Locality**										
Urban									2.698*	1.151
									[1.14,6.35]	[0.81,1.62]
Rural®										
State fixed effect	No	Yes	No	Yes	No	Yes	No	Yes	No	Yes
Log-likelihood	-1403.73	-1286.32	-1397.63	-1279.4	-1391.21	-1270.77	-1372.62	-1255.15	-1371.19	-1254.83
Likelihood Ratio chi2	8.04	5.23	20.24	19.07	33.08	36.33	70.27	67.57	73.12	68.21
Prob > chi2	0.0046	0.0222	0.0025	0.004	0.0009	0.0003	0.000	0.0000	0.000	0.0000

® Reference category; * p<0.05, ** p<0.01, *** p<0.001

In Table 4, we present the logistic regression analysis of the relationship between household sanitation facility and NMSV for rural Indian women. We find that those lacking private household sanitation have nearly two and half times the odds of NMSV (Model 1 OR = 2.45; 95% CI: 1.49–4.05) in our unadjusted model, with comparable effect sizes after adjusting for individual level demographics (Model 2 AOR = 2.49; 95% CI: 1.54–4.07). After models included household indicators of socio-economic status (Model 3 AOR = 2.01; 95% CI: 1.20–3.79) and women's employment (Model 5 AOR = 1.94; 95% CI: 1.16–3.62), those households lacking private sanitation have two times the odds of NMSV. In Table 4, we also ran the model with state fixed effects and found that this association remained significant, households lacking household sanitation have 1.6 times the odds of NMSV when controlling for the overall climate of the Sate. Employed relative to unemployed women were also significantly more likely to report recent NMSV. In our test of goodness of fit, we found that each successive model improved significantly from the previous model.

**Table 4:** Results of adjusted logistic regression model showing association between Indian rural women's household sanitation facility and th*eir e*xperience of NPSV within the last twelve months (2015–16), N = 52,475.

	Model-1		Model-2		Model-3		Model-4	
	OR		AOR		AOR		AOR	
***Predictor***								
***Household sanitation facility***								
Yes®								
No	2.456***	1.828**	2.486***	1.794**	2.012*	1.661*	1.941*	1.615*
	[1.46,4.11]	[1.21,2.75]	[1.49,4.13]	[1.17,2.74]	[1.12,3.58]	[1.04,2.63]	[1.09,3.44]	[1.01,2.56]
***Individual level predictors***								
***Age***								
15-24			0.692	1.022	0.667	1.006	0.844	1.199
			[0.30,1.55]	[0.58,1.79]	[0.29,1.51]	[0.57,1.77]	[0.38,1.86]	[0.67,2.11]
25-34			0.918	1.225	0.887	1.209	0.944	1.267
			[0.47,1.75]	[0.80,1.85]	[0.46,1.70]	[0.79,1.83]	[0.49,1.80]	[0.83,1.92]
35–49®								
**Education**								
Up to primary			0.879	1.174	0.68	1.127	0.674	1.15
			[0.32,2.40]	[0.56,2.45]	[0.25,1.82]	[0.51,2.46]	[0.25,1.78]	[0.52,2.51]
Secondary			0.756	0.887	0.679	0.906	0.719	0.984
			[0.31,1.81]	[0.44,1.77]	[0.28,1.60]	[0.44,1.83]	[0.30,1.69]	[0.48,1.99]
Higher®								
***Marital status***								
Never Married*®*								
*Ever married women*			0.609	0.423**	0.641	0.426**	0.694	0.456**
			[0.29,1.27]	[0.25,0.71]	[0.30,1.33]	[0.25,0.72]	[0.33,1.42]	[0.27,0.77]
***Household level predictors***								
***Caste***								
								
Scheduled castes					1.269	1.27	1.133	1.195
					[0.56,2.83]	[0.70,2.29]	[0.50,2.57]	[0.66,2.16]
Scheduled tribes					1.197	1.053	0.997	0.971
					[0.39,3.63]	[0.54,2.03]	[0.33,3.01]	[0.50,1.88]
Other backward class					0.817	1.023	0.79	1.026
					[0.38,1.74]	[0.58,1.77]	[0.36,1.69]	[0.58,1.78]
Others®								
***Religion***								
Hindu®								
Muslim					0.663	0.696	0.721	0.705
					[0.27,1.61]	[0.32,1.49]	[0.29,1.74]	[0.32,1.51]
Others					1.372	1.221	1.289	1.225
					[0.60,3.12]	[0.54,2.73]	[0.57,2.90]	[0.55,2.71]
**Household water facility**								
Within or nearby premises®								
Away from premises					1.382	1.457	1.307	1.422
					[0.70,2.71]	[0.89,2.38]	[0.66,2.56]	[0.86,2.32]
**Wealth quintile (without sanitation variable)**								
Poor					1.611	1.059	1.489	0.953
					[0.90,2.86]	[0.60,1.87]	[0.83,2.65]	[0.53,1.69]
Middle					0.922	0.681	0.84	0.646
					[0.49,1.72]	[0.40,1.15]	[0.45,1.56]	[0.37,1.10]
Rich								
***Working status of women***								
No®								
Yes							2.691***	2.222***
							[1.70,4.25]	[1.55,3.17]
State fixed effect	No	Yes	No	Yes	No	Yes	No	Yes
Log-likelihood	−968.91	−889.8	−962.42	−882.42	−956.23	−877.12	−943.99	−867.81
Likelihood Ratio chi2	12.77	8.85	25.74	23.60	38.12	34.21	62.60	52.83
Prob > chi2	0.0004	0.0029	0.0002	0.0006	0.0005	0.0019	0.000	0.0000

® Reference category; *p < 0.05, **p < 0.01, ***p < 0.001.

In Table 5, we present the logistic regression analysis of the relationship between household sanitation facility and NMSV for urban Indian women. As with our all India sample, we found no significant association between women's access to a household sanitation facility and experience of NMSV. In Table 5, when controlling for state fixed effects, we also found no significant association for urban Indian women. In the full model (Model 4, Table 5), urban women in households with water more than 30 min roundtrip from the household and employed urban women were at increased risk for NMSV. However, women with primary education were at lower odds of reporting recent NMSV.

**Table 5:** Results of adjusted logistic regression model showing association between Indian urban women's household sanitation facility and their experience of NPSV within the last twelve months (2015–16), N = 22,223.

	Model-1		Model-2		Model-3		Model-4	
	OR		AOR		AOR		AOR	
***Predictor***								
***Household sanitation facility***								
Yes®								
No	0.806	0.971	0.946	0.98	0.767	0.772	0.719	0.736
	[0.20,3.15]	[0.55,1.70]	[0.23,3.83]	[0.54,1.76]	[0.19,3.06]	[0.39,1.50]	[0.17,2.93]	[0.37,1.43]
***Individual level predictors***								
***Age***								
15-24			1.992	1.131	1.959	1.066	2.699	1.301
			[0.41,9.63]	[0.50,2.51]	[0.46,8.27]	[0.47,2.37]	[0.68,10.69]	[0.58,2.91]
25-34			0.836	0.832	0.8	0.782	0.899	0.859
			[0.31,2.24]	[0.44,1.55]	[0.33,1.94]	[0.41,1.46]	[0.37,2.13]	[0.45,1.61]
35–49®								
**Education**								
Up to and including primary			0.281	1.361	0.268	1.221	0.276	1.281
			[0.04,1.88]	[0.53,3.45]	[0.04,1.63]	[0.44,3.32]	[0.047,1.60]	[0.46,3.51]
Secondary			1.325	2.043	1.288	2.032	1.445	2.291*
			[0.33,5.18]	[0.94,4.42]	[0.37,4.47]	[0.92,4.48]	[0.42,4.90]	[1.02,5.09]
Higher®								
***Marital status***								
Never Married*®*								
*Ever married women*			1.448	0.777	1.413	0.768	1.661	0.818
			[0.56,3.71]	[0.35,1.71]	[0.58,3.40]	[0.34,1.69]	[0.69,3.99]	[0.37,1.79]
***Household level predictors***								
***Caste***								
								
Scheduled castes					0.261*	0.526	0.235*	0.509
					[0.07,0.97]	[0.23,1.16]	[0.06,0.86]	[0.23,1.12]
Scheduled tribes					3.426**	1.298	3.241*	1.233
					[1.41,8.29]	[0.54,3.07]	[1.26,8.31]	[0.52,2.92]
Other backward class					0.283*	0.386**	0.280*	0.382**
					[0.08,0.89]	[0.20,0.74]	[0.08,0.87]	[0.19,0.73]
Others®								
***Religion***								
Hindu®								
Muslim					0.643	0.797	0.675	0.818
					[0.09,4.44]	[0.37,1.72]	[0.09,4.73]	[0.37,1.77]
Others					0.665	2.910*	0.639	2.737*
					[0.26,1.68]	[1.24,6.80]	[0.24,1.63]	[1.17,6.40]
**Household water facility**								
Within or nearby premises®								
Away from premises					1.229	3.268**	1.292	3.210**
					[0.37,4.06]	[1.41,7.57]	[0.39,4.20]	[1.38,7.44]
**Wealth quintile (without sanitation variable]**								
Poor					1.071	2.161	0.894	1.945
					[0.34,3.36]	[0.80,5.77]	[0.27,2.86]	[0.72,5.21]
Middle					1.796	1.457	1.658	1.362
					[0.85,3.79]	[0.78,2.71]	[0.75,3.65]	[0.72,2.54]
Rich								
***Working status of women***								
No®								
Yes							2.776***	2.224**
							[1.75,4.39]	[1.29,3.81]
State fixed effect	No	Yes	No	Yes	No	Yes	No	Yes
Log-likelihood	−432.39	−355.75	−428.59	−352.15	−420.76	−338.54	−415.7	−334.57
Likelihood Ratio chi2	0.00	0.01	7.59	7.20	23.27	34.42	33.38	42.38
Prob > chi2	0.9715	0.9176	0.2695	0.3024	0.0561	0.0018	0.004	0.0002

® Reference category; *p < 0.05, **p < 0.01, ***p < 0.001.

## Discussion

Findings from this study demonstrate that women in rural India who lack a private household sanitation facility are more likely to have had a recent experience of NMSV, relative to rural women with private household sanitation facilities. These findings persist after accounting for demographics including age and marital status, socio-economic factors related to marginalization (e.g., caste, wealth), and factors in the state, suggesting that it may in fact be a consequence of heightened vulnerability when outside the home, which is consistent with prior qualitative evidence from India (Sahoo et al., 1982). Findings related to increased risk for NMSV among employed women, in both rural and urban settings, further reinforce indications that women requiring mobility in public spaces are at increased risk for NMSV in India (Raj et al., 2020). Importantly, such risks are not highly prevalent, as less than 1% of women in this study indicate NMSV in the past 12 months. Nonetheless, these findings are staggering in that they demonstrate, at a population level, that women's sexual safety is compromised simply by being in public spaces, either for purposes of biological need-due to lack of sanitation facilities, or for economic participation such as employment. Further, while we appreciate the elevated risk yields a small proportion of affected women, there remains a population level effect. Where almost 2 in 1000 rural women with sanitation facilities reports past year NMSV, more than 4 in 1000 rural women without sanitation facilities have experienced this violence in the past year.

Contrary to prior quantitative research from India on this issue (Jadhav et al., 2016), we did not find associations between household sanitation and NMSV for the nation as a whole, or for urban India. Differences may indicate improvements in urban areas over the past decade in private household sanitation access. Improvements in latrine access have been greater for urban relative to rural India in the period between these surveys (2005–06 and 2015–16) (Anuradha et al., 2017, pp. 107–110; International Institute for Population Sciences IIPS and ICF, 2017; WHO UNICEF 2017). Our findings related to effects seen for rural but not urban women may be attributable to locations of greater seclusion for open defecation in rural compared to urban areas (Saleem et al., 2019) and greater access to public latrine facilities in urban areas (Heijnen et al., 2014; O'Reilly et al., 2017). Again, this may be an improvement seen in the past few years as part of recent government sanitation schemes (Garn et al., 2017; International Institute for Population Sciences IIPS and ICF, 2017). Absence of findings for urban areas should not be taken to mean there is less need for focus in urban settings. Prior research suggests an association between sanitation access and sexual violence in urban slums (Kulkarni et al., 2017). Additionally, beyond issues related to sanitation, NMSV remains higher in urban relative to rural settings, and disproportionately affects younger women, certain marginalized castes, and employed women, indicating that demographic profile as well as mobility are affecting risk in urban settings (Raj et al., 2020).

Sexual violence against women is common worldwide and there have been numerous instances of sexual violence and murder of young women in India (World Health Organization, 2013; Menon & Allen, 2018; Raj & McDougal, 2014). Most sexual violence in India occurs in marriage; we know less about NMSV due to relatively low reporting (World Health Organization, 2013; Raj & McDougal, 2014). Prior research, not specific to India, has found greater risk for sexual violence among women living with economic deprivation (World Health Organization, 2013) and poorer development contexts (McDougal et al., 2018). We believe this study contributes to the research by shedding light on ways to reduce NMSV and is important to the goal of safety for women and girls in India (Ellsberg et al., 2015). Additionally, while previous WaSH research has focused, overwhelmingly, on the health benefits of improved vs. unimproved sanitation, our research points to the safety and security benefits for women of a private household sanitation facility that is not shared with other households. Furthermore, household sanitation reduces the psychosocial stress associated with open defecation and the use of shared sanitation (Sahoo et al., 1982) or the phsycial health risks of waiting to urinate or defecate alone or with someone they know (Kulkarni et al., 2017). These findings correspond with a mathematical modeling study of the value of optimal social investment in toilets in Khayelitsha, South Africa, predicated on an observed association between latrine access and sexual assault in that context. This study found that improving access to toilets in urban settlements would reduce sexual assault in the country (Gonsalves et al., 2015). Such an approach may have value in India, as well. However, we must be cautious in terms of building solutions for prevention of sexual violence that focus on limiting women's need to be in public, rather than focusing on the climate of acceptability of sexual harassment and assault, and victim-blaming, that persists in India and globally (Raj et al., 2020). Private household sanitation facilities offer some protection; but further efforts are needed in India to ensure that women have freedom of movement for full participation in society (Raj et al., 2020).

There are some limitations to our study. These limitations point to areas for possible future research. This is an observational study and there is a risk of bias. In addition, this study involves cross-sectional analyses, and thus we cannot assume causality. Furthermore, we rely on self-reported outcomes of NMSV, which may be underreported (McDougal et al., 2018; Palermo et al., 2014). While this analysis is limited to India, further analysis across countries on the relationship between household sanitation and NMSV could increase the generalizability of the results. Furthermore, once NFHS-5, 2018–2019, is released, it will be possible to analyze the data to see if these trends continue. In addition, there is a limited number of cases available in certain categories where we use multiple variables as covariates and our low prevalence outcome of interest. While it would be helpful to tease out the perpetrator of every incident of NMSV, this information is not available in the current data set, nor is the location or place where the NMSV occurred, or the total number of incidents or perpetrators of each incident of NMSV. Finally, we know of no national data, in India or elsewhere, that includes these data, and recommend that future surveys capture this information as well as the specifics on NMSV mentioned previously. This analysis, however, contributes to, and aims to reduce the silos in, the wider discussions on women's safety, gender-based violence and sanitation and furthers our knowledge on the risk factors associated with NMSV.(Rutstein & Johnson, 2004; Breiding et al., 2017; Abrahams et al., 2014).

## Conclusion

India is making large efforts to increase access to sanitation, specifically toilets, to reduce disease but also to improve gender equality, the latter achieved by increasing education and employment opportunities by ensuring women access to toilets in these settings (Duflo et al., 2015, p. 21521; Garn et al., 2017). Findings from this study extend this work by indicating that private household sanitation too may have value in reducing women's risk for non-marital violence, at least in rural India. We found that rural Indian women without a private household sanitation facility have greater odds of experiencing NMSV in the past year, relative to those with a private household sanitation facility that is unshared with other households. These findings remain significant even when we control for the overall climate of each state. These findings have important implications for policies related to sanitation and gender-based violence. While sanitation research and development has focused on the reduction of infectious diseases, further research and programming are needed that focus on improving sanitation access so that it is safe and gender-sensitive. As India takes steps toward improving access to sanitation and gender equality, rural areas have some of the greatest need for sanitation infrastructure and the positive externalities associated with access to private household sanitation. Additionally, as sanitation facilities are constructed, consideration should be given to the human right to access to water and sanitation for all people and freedom from violence. To this end, women and girls could participate in sanitation technology development, placement, maintenance and management to enhance the health and psychosocial benefits of sanitation and ensure its safety, security, and sustainability for all people, including women and girls (Hirai et al., 2016; Kayser et al., 2019). We know that the gender and health gains from sanitation interventions are affected by the type of sanitation technology selected, location of the sanitation, uptake, maintenance of the technology and sustained use (Anuradha et al., 2017; Clasen et al., 2014; Swain & Pathela, 2016). The inclusion of women and girls in decisions around sanitation location, design, operation and maintenance may help to both increase access to sanitation facilities and sustain use of the sanitation (Hirai et al., 2016; Kayser et al., 2019). Simultaneously, however, we cannot rely on private household sanitation access to reduce women's risk for sexual violence, as such an approach maintains perspectives of restricted mobility as a means of protection rather than accountability for perpetrators of violence. More work is needed on prevention of sexual violence through changes in social norms and interventions with potential and prior perpetrators. Improving access to sanitation in rural India is needed and this research suggests, it may help to reduce gendered based violence, NMSV specifically, and further enhance the health and rights-based benefits that sanitation offers all people, including women and girls (Prüss-Ustün et al., 2019; Jain et al., 2020; United Nations, 2010; Meier et al., 2013).

## Author Statement

G. Kayser's role was conceptualization, project administration, methodology, writing original draft, writing of subsequent drafts, review and editing, P. Chokhandre's role was formal analysis, writing original and subsequent drafts. N. Rao's role was in methodology development, writing-review and editing. L. McDougal's role was in methodology, writing-review and editing. Abhishek Singh's role was oversight of data collection design and methodology, contribution to research methodology, oversight of analysis, supervision, writing-editing, A. Raj's role was funding acquisition, conceptualization, methodology, supervision, writing-review, editing.

## Ethical statement

All authors accept responsibility for the content of this manuscript. This manuscript is an original work, has not been previously published elsewhere, whole or in part, and is not under consideration for publication elsewhere.

## Financial disclosure

AR received funding from the 10.13039/100000865Bill and Melinda Gates Foundation (Grant number: OPP1179208) for this research (https://www.gatesfoundation.org). The funders had no role in study design, data collection and analysis, decision to publish, or preparation of the manuscript.

## Declaration of competing interest

We declare no competing of conflicts of interests.
